# Relationship between Glycemic Levels and Treatment Outcome among Critically Ill Children admitted into Emergency Room in Enugu

**DOI:** 10.1186/s12887-017-0879-8

**Published:** 2017-05-16

**Authors:** Nwachinemere Davidson Uleanya, Elias Chikee Aniwada, Ikenna Chidiebele Nwokoye, Ikenna Kingsley Ndu, Christopher Bismarck Eke

**Affiliations:** 1 Department of Pediatrics, Enugu State University Teaching Hospital, Enugu, Nigeria; 2 Department of Community Medicine, University of Nigeria Teaching Hospital, Ituku-Ozalla, Enugu, Nigeria; 3 Department of Pediatrics, Federal Teaching Hospital, Abakaliki, Nigeria; 4 Department of Pediatric, University of Nigeria Teaching Hospital, Ituku-Ozalla, Enugu, Nigeria

**Keywords:** Critically ill, Children, Hypoglycemia, Hyperglycemia, Treatment outcome

## Abstract

**Background:**

Critically ill children are those in need of immediate attention on arrival to an emergency room. The importance of glycemic level measurement as well as maintaining the patency of the airway, effective breathing and circulation cannot be overemphasied. It has been highlighted that the peak hyperglycemia and hypoglycemia predict poor prognosis, longer lengths of hospital stay and higher mortality. The study aims to assess the relationship between glycemic level and treatment outcomes as well as length of hospital stay.

**Methods:**

Analytical cross sectional method was used to study critically ill children aged ≥1 month to ≤10 years admitted into the Children Emergency Room of Enugu State University Teaching Hospital, Enugu. Their admission blood glucose was done. Interviewer administered questionnaire was used to collect information including sociodemographics, duration of hospitalization and outcome of treatment. Data was analysed using SPSS version 20. Chi square, logistic regressions and Kruskal Wallis tests were done as appropriate.

**Results:**

A total of 300 patients were recruited. One hundred and seventeen (39%) had hyperglycemia, 62 (20.7%) patients had hypoglycaemia and 121 (40.3%) had euglycemia. Two hundred and fifty two (84%) were discharged while 48 (16%) died. There was significant association between glycemic levels and treatment outcome (*p* = < 0.001). Among the 48 who died, 12 (25.0%) had euglycemia, 21 (43.75%) had hypoglycaemia while 15 (31.25%) had hyperglycemia. On multivariate analysis, there was statistically significant association between hypoglycaemia and mortality (*p* = < 0.001). Unadjusted, those children with hypoglycaemia at presentation were about 4.7 times (UOR = 0.21, 95% Cl: 0.08–0.38) and adjusted, about 5 times (AOR = 0.20, 95% CI: 0.09–0.47) less likely to survive compared with those with euglycemia. Although not statistically significant, those with hyperglycemia were about 1.3 times less likely to survive compared with euglycemic children, adjusted and unadjusted (UOR = 0.75, 95% Cl: 0.33–1.68).

**Conclusion:**

While both hypo- and hyperglycemia are associated with mortality, hypoglycaemia had a greater effect than hyperglycemia. Glycemic levels significantly affects treatment outcome.

**Electronic supplementary material:**

The online version of this article (doi:10.1186/s12887-017-0879-8) contains supplementary material, which is available to authorized users.

## Background

Critically ill-children are those children in need of immediate attention on arrival to a health facility. This constitutes a heterogeneous group of acutely ill children such as those convulsing, unconscious, cyanosed, lethargic or floppy [1, 2]. Others are those with severe chest indrawing, dehydration (severe), petechiae or purpura, stridor, hypoxemia, hypothermia and hyperpyrexia [1–3]. These symptoms cover most serious medical cases presenting at the emergency rooms, including acute respiratory infections (ARIs), febrile convulsions, diarrheal diseases, severe malaria, meningitis, sepsis and septic shock, burns etc.

In these children, therefore as much as it is essential to maintain the patency of the airway, effective breathing and circulation, it is also important that the glycaemia level should be assessed. It has been highlighted that either hypoglycaemia or hyperglycaemia is substantially associated with increased morbidity and mortality in diabetic and nondiabetic critically ill children and adults [3, 4]. In particular, both the peak hyperglycaemia and duration of hyperglycemia have been found to be poor prognostic factors, predict longer lengths of hospital stay and mortality in the diabetic and nondiabetic adults [4, 5]. It then means that the more severe the hyperglycaemia, the more risk of mortality [6]. Hypoglycemia is also very common among nondiabetic critically ill children. It is an independent risk factor for increased mortality rates and worsening organ function [4, 6]. This risk increases the more severe the hypoglycaemia [4].

The human body, in an otherwise healthy state is able to maintain a tight glycemic levels independent of ingested food or energy expenditure. However, any form of stress that follows critical states may lead to profound impairments in this homeostasis [7].

Hyperglycaemia represents an extreme form of ‘stress’ in critically ill children. Initially, stress induced hyperglycemia during critical illness was thought to be an adaptive response that was either unimportant or improved survival [8]. However, it is no longer considered a physiological or benign condition as it results from a surge of endogenous counter-regulatory hormones, insulin resistance and other diabetogenic factors with the release of pro-inflammatory mediators, oxidative stress and therapeutic interventions such as exogenous dextrose infusion, glucocorticoids, vasopressors like dopamine, β- blockers, antibiotic solutions, overfeeding and bed rest [9–11]. These interfere with insulin receptor signalling and/or insulin-regulated glucose channels and directly interfere with proper glucose transport and utilization in peripheral cells. Hyperglycemia in these children therefore is due to the combined effects of insulin resistance, glucose intolerance, increased gluconeogenesis, counter-regulatory hormone release(epinephrine, norepinephrine, glucagon, cortisol, growth hormone) and cytokines (tumor necrosis factor-α [TNF-α], interleukin-1 [IL-1], interleukin-6 [IL-6]) [8, 10, 12]. Studies have suggests that the degree and duration of hyperglycemia is related to the severity of the disease and is an important prognostic marker [8, 12]. The consequences of hyperglycemia are protean, including neutrophilic dysfunction, decreased intracellular bactericidal and opsonic activity, damaged mitochondrial proteins, modification of the innate immune system and impairment of endothelial function [9, 13–18]. However, it is still not very clear whether hyperglycemia is a marker of critical illness in children or an etiological factor contributing to worse outcome. Hyperglycemia in children may have different effects on morbidity and mortality compared with adults as a consequence of different metabolic demands, differences in co-morbid conditions or age-dependant factors [11].

On the other hand, hypoglycemia is equally a common metabolic feature occurring in critically ill children. The signs may be subtle but nonspecific. The cause is not well understood but includes cytokine-induced impairment of gluconeogenesis, impaired counter-regulatory hormone response and depletion of glucose stores in starvation [9, 19–21]. Usage of glucose by the parasites may also contribute significantly to hypoglycaemia. During critical illness there is increased glucose utilization, inadequate nutrition, and decreased endogenous glucose production [22]. Hypoglycemia among critically ill children is more common among patients with medical conditions, female gender and mechanical ventilation [9]. It is a marker of disease severity [3, 6, 20]. Although the brain contains enzymes that can metabolize alternate sources of fuel (e.g., lactate and ketones) when arterial blood glucose falls below 54 mg/dl, cerebral metabolism and function decline [22]. A rapid detection and treatment of severe hypoglycaemia are critical to prevent harm to the brain, especially in critically ill children. This is because permanent cardiac and neurological effects of hypoglycaemia correlate with the duration of time that cerebral tissues are deprived of glucose [3, 6]. Although the brain can utilize other substrates, especially in newborns and during prolonged fasting, however, no substrate can successfully correct the neurophysiologic sequelae of neuroglycopenia [6]. Long term effects of hypoglycaemia in infants include decreased head size, lower intelligent quotient, and specific regional brain abnormalities [3].

It is then logical to say that critically ill-children are very susceptible to hypo- and hyperglycaemia. However, there are varied controversies as regards the definition of hypo- or hyperglycemia. The fact is that counter-regulatory hormones are activated at glucose levels of 65 mg/dl or less in nondiabetics [4, 23]. While some authorities accept 40 mg/dl as definition for hypoglycaemia among nondiabetic patients, < 45mgl/dl for neonate others define hypoglycaemia as blood glucose level of <60 mg/dl or ≤65 mg/dl while some report cut off values of <80 mg/dl [3, 4, 22–26]. The fact that the definition of hypoglycaemia in paediatrics varies depending on age and fasting state remains valid. Also, there have been no specific criteria for defining hyperglycemia among critically ill, nondiabetic children [4, 27–29].

In our centre, routine glycemic check at admission has not yet been adopted as a standard practice despite the potential risks associated with both hyperglycemia and hypoglycemia. Based on these, the study was then undertaken to find out the relationship between glycemic levels of critically ill children and duration of hospital stay as well as treatment outcome.

## Methods

This was an analytical cross sectional study carried out among critically ill children who were admitted into the Children Emergency Room of Enugu State University Teaching Hospital, Enugu. It currently serves as the only not-for-profit government owned emergency room in Enugu metropolis. Ethical clearance was obtained from the Enugu State University Teaching Hospital Health Research and Ethics Committee. Both written and oral informed consent was obtained from each parent(s)/caregiver and confidentiality maintained in the entire study. A total study was done involving 300 critically ill children from February 2014 to October 2015. All critically ill children ≥1 month to ≤10 years of age who presented with convulsion, unconsciousness, cyanosed, lethargic or floppy, those with severe chest indrawing, severe dehydration, petechiae or purpura, stridor, hypoxemia, hypothermia and hyperpyrexia, and had at least one blood glucose measurement on admission before commencement of intravenous fluid or antibiotics were consecutively recruited. All children in the study were studied from day of admission and followed up till discharge or death. Exclusion criteria include those children whose parent(s)/caregiver did not consent, all diabetics, all children who received intravenous fluid, steroids, vasopressors or antibiotics prior to presentation and all children who did not present with the features mentioned above. A structured interviewer questionnaire was administered to parent(s)/caregiver of each child. Some of the information sought by the questionnaire included: sociodemographics, duration of hospital stay and outcome of treatment. One microliter (1 μL) of whole blood was collected at each measurement and tested for blood glucose level using the Accu-chek active test strip and glucometer (Roche Diagnostics GmbH, Mannheim, Germany) on admission. Quality assurance followed standard hospital laboratory procedures and included a daily recheck of calibration and was repeated if the results varied by more than 1 mg/dl. The calibration was based on the Hexokinase method of spectrophotometry and the measuring interval is 10-600 mg/dl. Hence blood glucose level of ≤9 mg/dl read low and High if >600 mg/dl. The Accu-chek active blood glucose measurement has been validated in Nigeria and correlates positively with laboratory blood glucose measurement by spectrophotometry, *r* = 0.84, *p* = 0.05. Its sensitivity is 75% with a specificity of 99.8% relative to the gold standard. It also has a positive predictive value of 94.7% and a negative predictive value of 98.7% [30]. Glucose variability index was also calculated. This is an index which is calculated with at least 3 or more glucose measurements by dividing the absolute difference of sequential glucose measurements by the difference in collection time (in hours +0.01); with the mean of the ratios for each patient forming the variability index [4].

Those children with hypoglycaemia were immediately managed according to standard protocols. Our protocol calls for initial bolus infusion of 4mls/kg of 10% dextrose water either intravenously or intraosseously followed by 10% dextrose maintenance. Feeding was also commenced as soon as is feasible for the patient. As for those with hyperglycemia, they were commenced on 0.9% saline solution and their blood glucose monitored closely. All necessary care was given to each patient according to their needs. For the purposes of this study, we chose to define hypoglycaemia as blood glucose level of ≤65 mg/dl and hyperglycemia as ≥110 mg/dl. [4, 22] Our main outcome measures were mortality and discharged.

All data were analysed using SPSS software version 20. Tables were presented accordingly. Associations between socio-demographics with glycemic level and treatment outcome were determined using Chi-square test. Binary logistic regression was equally done to ascertain predictors of treatment outcome. *P* value at level of ≤0.05 was accepted as significant.

## Results

A total of 300 patients who met the inclusion criteria were studied. Of these151 (50.3%) were aged ≤1 year old while 149 (49.7%) were aged >1–10 years (median age: 12.4 months; IQR: 6.3–36.0 months). There were 187 boys (62.3%) and 113 (37.7%) girls and of these 95.0% were Igbos. Most of the children (81.7%) belonged to the lower class (Table 1).

**Table 1:** Socio-demographic characteristics of subject population

	Frequency *n* = 300	Percentage (%)
Age (Years)		
≤ 1	151	50.3
> 1 ≤ 10	149	49.7
Sex		
Male	187	62.3
Female	113	37.7
Tribe		
Igbo	285	95.0
Others^a^	15	5.0
Social class		
Upper	55	18.3
Lower	245	81.7

^a^Yoruba, Hausa/Fulani, Ibibio, Urhobo, Lebanese

The medical conditions (diagnoses) observed among the critically ill children is as shown in Table 2. Most were acute infectious diseases – severe malaria (29.7%), severe sepsis (25%), diarrheal diseases (19%) etc. The mean duration of hospitalization was 7.6 ± 5.8 days, with minimum hospital stay of one day and maximum of 28 days.

**Table 2:** Distribution of Diagnoses among the critically ill children

Diagnoses	Frequency *n* = 300	Percent %
Severe Malaria^a^	89	29.7
Sepsis^b^	75	25.0
Diarrheal diseases^c^	57	19.0
Respiratory diseases^d^	44	14.7
Meningoencephalitis	25	8.3
Others^e^	10	3.3

^a^Severe malarial anemia ± CCF, Cerebral malaria, Prostration, hypoglycemia, Jaundice, hemoglobinuria, hyperpyrexia,

^b^Sepsis, severe sepsis, Septic shock, Disseminated Intravascular Coagulation (DIC)

^c^Acute gastroenteritis, Hypovolemic shock, Enteric fever

^d^Bronchopneumonia, Bronchiolitis, Pharyngotonsilitis, Empyema thoracis, Acute otitis media

^e^Acute renal disease, Intestinal obstruction, seizure disorders

Hyperglycemia was prevalent among these critically ill children. One hundred and seventeen (39%) of the cases had hyperglycemia. Hypoglycemia was also found to be prevalent among pediatric emergency room patients. A total of 62 (20.7%) patients had hypoglycaemia while 121 (40.3%) had euglycemia. Of the 300 critically ill children, 252 (84%) were discharged while 48 (16%) died Table 3. Among the 48 who died, 12 (25.0%) patients had euglycemia, while 21 (43.75%) and 15 (31.25%) patients had hypoglycaemia and hyperglycemia respectively. Majority of the deaths 35 (72.9%) occurred in the emergency room within the first 72 h. Of these, 19 (54.3%) died within 24 h of presentation. Eleven of these had hypoglycaemia, 5 had hyperglycemia and 3 euglycemia. There was significant association between the duration of hospitalization and treatment outcome (χ^2^ = 63.92, *p* = 0.000).

**Table 3:** Distribution of glycemic level and treatment outcome

	Frequency *N* = 300	Percentage %
Glycemic level		
Euglycemia	121	40.3
Hypoglycemia	62	20.7
Hyperglycemia	117	39.0
Treatment Outcome		
Discharged	252	84.0
Died	48	16.0

On multivariate analysis, there was significant association between glycemic levels and treatment outcome (χ^2^ = 19.914, *p* = < 0.001). There was also statistically significant association between hypoglycaemia and mortality. Those children with hypoglycaemia at presentation were about 4.7 times less likely to survive compared with those with euglycemia (UOR = 0.21, 95% Cl: 0.08–0.38). Although not statistically significant, those with hyperglycemia were about 1.3 times less likely to survive compared with euglycemic children (UOR = 0.75, 95% CI: 0.33–1.68, AOR = 0.75, 95% CI: 0.33–1.7) unadjusting and adjusting for sociodemographics (Table 4). Adjusting for sociodemographics, those with hypoglycaemia were about 5 times less likely to survive compared with those with euglycemia (AOR = 0.20, 95% CI: 0.09–0.47). On further categorization of hyperglycemia into mild (110-149 mg/dl), moderate (150-199 mg/dl) and severe (≥ 200 mg/dl), there was statistically significant association between severity of hyperglycemia and treatment outcome. Those with severe hyperglycemia were about 8.24 times less likely to survive compared with those who had mild hyperglycemia.

**Table 4:** Predictors of treatment outcome

	Bivariate analysis			Multivariate analysis
	Died n (%)	Discharged n (%)	*p* value^a^	AOR (95% CI)^b^
Glycaemic level				
Euglycemia	12 (9.9)	109 (90.1)		
Hypoglycaemia	21 (33.9)	41 (66.1)	0.000	0.21(0.09–0.47)
Hyperglycaemia	15 (12.8)	102 (87.2)		0.75(0.33–1.7)
Age (years)				
≤ 1	26 (17.2)	125 (82.8)		
>1 ≤ 10	22 (14.8)	127 (85.2)	0.562	1.34(0.69–2.60)
Sex				
Female	28 (15.0)	159 (85.0)		
Male	20 (17.7)	93 (82.3)	0.533	0.74(0.39–1.43)
Tribe				
Igbo	47 (16.5)	238 (83.5)		
Others^d^	1 (6.7)	14 (93.3)	0.312	2.80(0.34–22.95)
Social class				
Upper	9(16.4)	46(83.6)	0.935	0.87(0.38–2.01)
Lower	39(15.9)	206(84.1)		
				UOR(95% CI)^c^
Glycaemic level				
Euglycemia	12 (9.9)	109 (90.1)		
Hypoglycaemia	21 (33.9)	41 (66.1)	0.000	0.21(0.10–0.48)
Hyperglycaemia	15 (12.8)	102 (87.2)		0.75(0.33–1.68)
*Hyperglycaemia*				
*mild*	*4(6.3)*	*60(93.8)*		
*moderate*	*5(13.9)*	*31(86.1)*	*0.006*	*0.41(0.10–1.65)*
*severe*	*6(35.3)*	*11(64.7)*		*0.12(0.03–0.51)*

^a^ *P* value on bivariate analysis

^b^Adjusted odds ratio (95% confidence Interval) on multivariate analysis

^c^unadjusted odds ratio of univariate analysis

^d^Yoruba, Hausa/Fulani, Ibibio, Urhobo, Lebanese

Using Kruskal Wallis test, there was no statistically significant association between glycaemic levels and duration of hospitalization (*p* = 0.903).

## Discussion

Hyperglycemia is a very prevalent condition among critically ill nondiabetic patients – occurring in 39% of our patients. Though, several mechanisms including cytokine production, acute dyslipidemia and endothelial dysfunction, accelerated glucose toxicity that leads to metabolic disturbances, hypercoagulation and increased cellular apoptosis have all been proposed as possible causes [4, 31]. The prevalence of hyperglycemia in this study is smaller than that of Wintergerst et al [4] and Faustino et al. [5] respectively. This may be attributed to the larger sample size in their study and the fact that surgical patients were included in their study, with the surge in glucose levels following stress, contributing significantly to the higher prevalence in their study. It is also smaller than that documented by Ameyaw, et al. [22] who did not study critically ill children alone. However, in this study, it was observed that as hyperglycemia increases, there was an increased significant association with mortality. This agrees with the findings of Wintergerst, Srinivasan and Patki [4, 11, 29], suggesting the toxic effect of glucose (hyperglycemia) in critical illness and identifies it as a predictor of mortality among children with medical conditions. Almost a third of the mortality in this study had hyperglycemia, a third of which died within 24 h of presentation in the Emergency room. Those children with hyperglycemia on presentation were slightly more likely to die than be discharged. This is worse with higher concentrations of hyperglycemia where the odds of death were about 8 times more likely in those with severe than in those with mild hyperglycemia.

Hypoglycemia also was common among the critically ill children, occurring in 20.7% (62) of the patients. This hypoglycaemia prevalence is comparable with that of Wintergerst, et al. [4] who recorded hypoglycemia prevalence of 18.6% at the same cut off among critically ill children and Onyiriuka, et al. [20] who recorded 18.3% among children with hypoglycaemia in Benin city. However, it is higher than that recorded by Ameyaw et al. [22] in Kumasi. This may be as a result of the value used to define hypoglycaemia in that study. Our result showed a strong association between hypoglycaemia and mortality. This agrees with other results among critically ill children [4, 6, 11, 22, 29]. Those children with hypoglycaemia at presentation were almost 5 times less likely to be discharged home. Most of these deaths associated with hypoglycaemia (11) in this study, occurred in the emergency room especially within the first 24 h of admission. This also agreed with the findings of Elusiyan, et al. [32] who found out that presence of hypoglycaemia at admission was associated with death and dying within 24 h of admission. In Kenya, Osier, et al. [33] found that mortality for children with abnormal blood glucose was 34.2% compared to 7.6% in euglycaemic children in an Emergency ward. Among those that died in this study, 43.75% had hypoglycemia signifying the potent lethal effect of hypoglycemia among critically ill children.

Overall, in this study, glucose variability had the strongest association with mortality (*p* = < 0.001). This also agreed with other studies [4, 22]. Wingerst, et al. [4] found that there is a strong association between mortality and highest or lowest blood glucose level. Glucose variability has been shown to indirectly lead to increased oxidative stress, resulting in direct cellular damage and apoptosis [4, 33, 34]. This follows the increased production of reactive oxygen species such as superoxide and peroxynitrite. These has been postulated to be the mechanism for glucose induced vascular damage [4, 33].

Majority of the mortality in this study occurred in the Emergency room, especially within the first 24 h where 54.3% of these deaths occurred. Our study showed that there was significant association between duration of hospitalization (within first 72 h) and treatment outcome. The risk of death is markedly reduced once a patient is able to survive the first 3 days in Emergency room.

This study however, did not find any significant association between duration of hospital stay and glycemic levels. This differs from that of some other studies [4, 11, 35].

## Conclusion

Both hyperglycemia and hypoglycaemia are very prevalent among critically ill children. While both are associated with mortality, hypoglycaemia had a greater association with mortality than hyperglycemia. Glucose variability are also significantly associated with treatment outcome. It is hoped that the findings of this study will draw more attention to the need for routine glycemic check at admission thus reducing morbidity and mortality from hyperglycemia and hypoglycemia.

### Limitations

Lack of adjustment for nutritional status of children and prior determination of the risk of mortality using PRISM/PIM.

## Additional files

Additional file 1: BMCPed glycemic levels and treatment outcome data. (XLS 158 kb)

Additional file 2: BMCPed data on Hypoglycemia and outcome1. (XLS 160 kb)

## Abbreviations

AOR: Adjusted odd ratio
ARI: Acute respiratory Infection
CI: Confidence Interval
IL: Interleukin
IQR: Interquartile range
SPSS: Statistical package for social sciences
TNFα: Tumor necrotic factor alpha
UOR: Unadjusted odd ratio
μL: Microliter
